# DMcloud: A Local Fitting Method for Accurate Structure Modeling in Medium to Low Resolution Cryo-EM Maps

**DOI:** 10.1063/4.0000885

**Published:** 2025-10-27

**Authors:** Genki Terashi, Xiao Wang, Yuanyuan Zhang, Han Zhu, Daisuk Kihara

**Affiliations:** 1 Purdue University, West Lafayette, Indiana, USA; 2 University of Washington, Seattle, Washington, USA

## Abstract

Cryogenic electron microscopy (cryo-EM) has become an essential method in structural biology for resolving large macromolecular assemblies. As cryo-EM continues to expand into more challenging targets, such as flexible assemblies and large macromolecular structures, the availability of medium to low-resolution maps (5–10 Å) has increased. However, interpreting these maps continues to be challenging due to the low quality of density features at limited map resolutions and inaccuracies in the predicted atomic models. While structure prediction methods such as AlphaFold (Abramson et al. 2024; Jumper et al. 2021) have led to significant improvements in model availability and quality, these models frequently suffer from domain misorientation, inaccurate flexible regions, or errors in complex formation. Consequently, traditional global fitting or rigid-body fitting approaches frequently struggle to achieve accurate model fitting, especially when large conformational changes or model errors exist in the predicted model.

To address these challenges, we developed DMcloud, a new method that performs local structure fitting to improve model accuracy in cryo-EM maps at 5–10 Å resolution. DMcloud is designed to address cases where AlphaFold2 (AF2) models contain accurate local domains but incorrect global orientations. By converting both the AF2 model and cryo-EM map into point clouds, DMcloud performs iterative local alignments and denoising to refine model placement. This approach enables the correction of domain orientation errors and improves overall model-map agreement. DMcloud builds on our earlier method, DiffModeler (Wang et al. 2024), which performs global fitting of protein complexes using diffusion-based backbone tracing and AlphaFold-guided model assembly. While DiffModeler performs global structure modeling, DMcloud provides a complementary solution focused on fine-grained local fitting.

We evaluated DMcloud on 71 intermediate- and 50 high-resolution cryo-EM maps using AF2 models from the AlphaFold Protein Structure Database. DMcloud outperformed traditional fitting methods, particularly in low-resolution cases and where AF2 models contained significant structural discrepancies. Figure 1 shows examples of modeling results by DMcloud for high and intermediate-resolution maps. Each row corresponds to a different target: a. EMD-20815, b. EMD-23192, c. EMD-21536, and d. EMD-12221 and shows (i) the EM density map, (ii) the reference PDB structure, (iii) a comparison between the DMcloud model and the reference PDB structure, and (iv) a comparison between the AlphaFold2 model and the reference PDB structure after structural alignment. As shown in Figure 1, DMcloud successfully corrected misoriented domains and reduced false model regions.

DMcloud offers an accurate, automated framework for cryo-EM model fitting and is particularly effective in challenging cases where predicted models contain local inaccuracies, such as domain misorientations or flexible regions. By focusing on local structure alignment using point cloud representations and iterative refinement, DMcloud enables more precise model placement than conventional global or rigid-body fitting approaches. Its ability to selectively identify and adjust only the well-supported regions of given structure models makes it useful for large assemblies and partially resolved complexes. The method is freely available through our web server at https://em.kiharalab.org, alongside other AI-based tools for cryo-EM maps.

